# The effects of Global Fund financing on health governance in Brazil

**DOI:** 10.1186/1744-8603-8-25

**Published:** 2012-07-16

**Authors:** Eduardo J Gómez, Rifat Atun

**Affiliations:** 1 Department of Public Policy & Administration, Rutgers University, 401 Cooper Street, Camden, NJ, USA; 2 International Health Management, Imperial College Business School and Faculty of Medicine, Imperial College London, Exhibition Road, SW7 2AZ, London, UK

## Abstract

**Objectives:**

The impact of donors, such as national government (bi-lateral), private sector, and individual financial (philanthropic) contributions, on domestic health policies of developing nations has been the subject of scholarly discourse. Little is known, however, about the impact of global financial initiatives, such as the Global Fund to Fight AIDS, Tuberculosis, and Malaria, on policies and health governance of countries receiving funding from such initiatives.

**Methods:**

This study employs a qualitative methodological design based on a single case study: Brazil. Analysis at national, inter-governmental and community levels is based on in-depth interviews with the Global Fund and the Brazilian Ministry of Health and civil societal activists. Primary research is complemented with information from printed media, reports, journal articles, and books, which were used to deepen our analysis while providing supporting evidence.

**Results:**

Our analysis suggests that in Brazil, Global Fund financing has helped to positively transform health governance at three tiers of analysis: the national-level, inter-governmental-level, and community-level. At the national-level, Global Fund financing has helped to increased political attention and commitment to relatively neglected diseases, such as tuberculosis, while harmonizing intra-bureaucratic relationships; at the inter-governmental-level, Global Fund financing has motivated the National Tuberculosis Programme to strengthen its ties with state and municipal health departments, and non-governmental organisations (NGOs); while at the community-level, the Global Fund’s financing of civil societal institutions has encouraged the emergence of new civic movements, participation, and the creation of new municipal participatory institutions designed to monitor the disbursement of funds for Global Fund grants.

**Conclusions:**

Global Fund financing can help deepen health governance at multiple levels. Future work will need to explore how the financing of civil society by the Global Fund and other donors influence policy agenda-setting and institutional innovations for increased civic participation in health governance and accountability to citizens.

## Introduction

In recent years, a rise in the international financing of health programmes in developing nations have spurred scholars and policy-makers working on global health policy to undertake studies to better understand the effects of these initiatives [1-12]. In particular, a growing body of work has explored how new international funding has benefited health outcomes, influenced policy-making [4,13-15], affected the allocative efficiency and equity of international financing [3,10,12,13,16], as well as the additionality of new financing [1]. Others have explored the positive policy synergies created by external investors, that is, donors (such as national governments through bilateral assistance), corporations, individuals (philanthropic), and global financial initiatives (such as the Global Fund to Fight AIDS, Tuberculosis, and Malaria), on health systems, the unintended consequences of these investments [1,6,17-19], and their effects on the design of service delivery [6,12,18-24].

An area that has also attracted interest but where empirical evidence is all but absent relates to the extent to which these external investments have enhanced local institutional capacity [8,11,20]. Additionally, few studies have examined how external investments have increased government commitments to combating relatively neglected diseases through inclusive and participatory approaches that foster a closer working relationship between policy-makers and civil society.

Indeed, while the influence of external investments on domestic policy-making, financing, and service delivery has received scholarly attention, the influence of these investments on health governance has been less explored. We address this gap in the literature while providing new insights into how external investments strengthen health governance at multiple levels. We elucidate a new multi-stakeholder approach to health governance, where external investors, such as global financial initiatives, are used to both strengthen and catalyse health governance. The multi-stakeholder approach we describe encapsulates national intra-governmental (*i.e.*, within and between agencies), inter-governmental (*i.e.*, between national and sub-national agencies), governmental-civil societal, and community-level processes and responses to disease: more specifically, government leadership, policy commitment, and agency cooperation (intra-governmental), inter-governmental cooperation between national, state, and municipal health agencies [25,26], as well as national bureaucratic cooperation with civil society, local community ownership, and community mobilization to achieve common health policy goals [25,26]. Empirically, this study examines the case of Brazil, where a limited set of studies have explored the influence of national and sub-national bureaucratic stewardship, community partnerships, and participation in enhancing community-level prevention programmes [27], as well as civic mobilization in enhancing government responses to health [28].

Nevertheless, to date no studies have analysed how global financial initiatives, such as the Global Fund to Fight AIDS, Tuberculosis, and Malaria (henceforth, Global Fund), have influenced this process in Brazil, as well as government commitment to combating relatively neglected diseases, such as tuberculosis (TB). Analyzing the Global Fund is particularly important given its policy commitment to country ownership, multi-stakeholder participation and inclusiveness in its governance structure through civic participation in the policy-making processes *via* Country Coordinating Mechanisms (CCMs), its Board and committees and dual track financing that enables direct financing of non-state actors as grant recipients [5,18,29-31]. While multilateral lenders, country donors, and global financial initiatives, such as the World Bank, President’s Emergency Plan for AIDS Relief (PEPFAR), and GAVI (Global Alliance for Vaccines and Immunisation), respectively, have positively influenced governance, decentralization and healthcare services management, as well as civic mobilization in Brazil for other heath and infrastructural issues – mainly HIV/AIDS [32,33], the Global Fund’s contributions to this process has not received sufficient scholarly attention. This study therefore examines the impact of Global Fund investments at several tiers of health governance in Brazil: the national intra-governmental, inter-governmental, government-civil societal and community-level.

**Figure 1:**
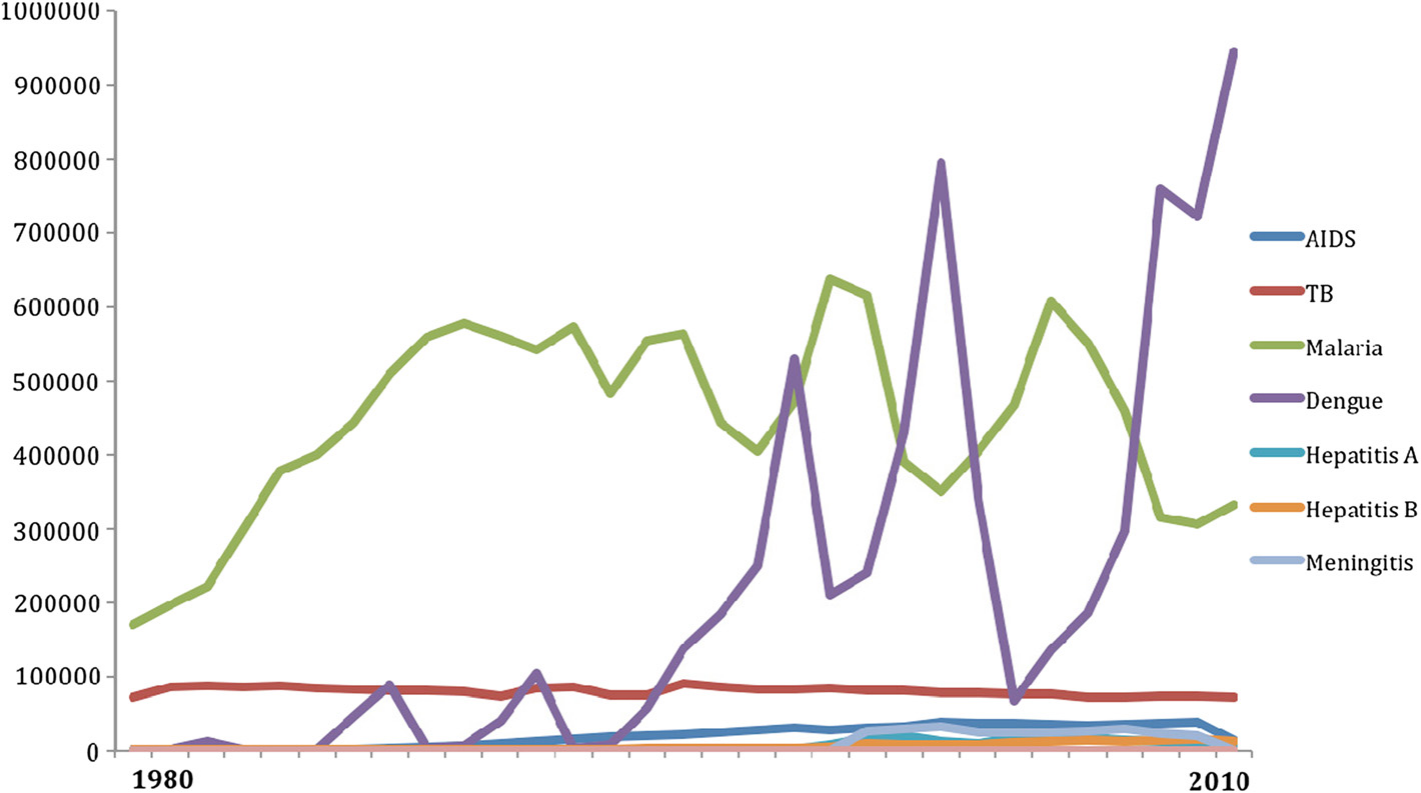
Brazil: number of new cases of key infectious diseases 1980-2010.

As Figure 1 and Table 1 illustrates, when compared to other diseases, TB has posed a particularly high burden in Brazil, ranking third behind dengue and malaria in terms of yearly reported cases from 1980 to 2010, followed by AIDS in fourth place. Several other commonly known diseases, such as hepatitis A & B, meningitis and yellow fever rank much lower. In a context of increasingly scarce funding for healthcare and the need to respond to multiple diseases [34], the Global Fund’s support has helped the MOH respond to TB.

**Table 1:** Brazil: number of new cases of key infectious diseases 1980-2010

	**AIDS**	**Tuberculosis**	**Malaria**	**Dengue**	**Hepatitis A**	**Hepatitis B**	**Meningitis**	**Yellow Fever**
1980	1	72608	169871	na	na	na	na	25
1981	0	86411	197149	na	na	na	na	22
1982	10	87822	221939	11000	na	na	na	24
1983	41	86617	297687	na	na	na	na	6
1984	134	88366	378257	na	na	na	na	22
1985	554	84310	399462	na	na	na	na	7
1986	1183	83731	443627	46309	na	na	na	9
1987	2848	81826	508864	88407	na	na	na	16
1988	4598	82395	559535	1570	na	na	na	26
1989	6327	80375	577520	5367	na	na	na	9
1990	9092	74570	560396	39322	na	na	na	2
1991	12135	84990	541927	104398	na	na	na	15
1992	15221	85955	572933	1658	na	na	na	12
1993	17452	75453	483367	7388	na	na	na	83
1994	19189	75759	555135	56584	639	1275	na	19
1995	21980	91013	564570	137308	1229	1423	na	4
1996	25067	85860	444049	183762	915	1672	na	15
1997	27572	83309	405051	249239	672	2028	na	3
1998	30506	82931	471894	528388	1649	1831	na	34
1999	28010	84337	637474	209688	8210	4204	na	76
2000	31493	81862	615247	239870	16661	7537	26931	85
2001	31649	81182	389775	428117	20671	7111	30072	41
2002	37902	77836	349965	794219	12907	6239	33212	15
2003	37619	78606	405017	341776	8308	6352	25431	64
2004	36834	77694	466439	66,000	17448	10246	25375	5
2005	36009	76468	607801	135,000	21580	12015	25925	3
2006	34614	72213	550917	185,000	17021	12134	27543	2
2007	35351	71825	458649	296,000	13301	12049	29935	13
2008	37465	73531	315630	758051	11629	13147	23623	46
2009	38538	73082	306908	721546	10743	14468	21141	47
2010	13520	71790	333429	944662	5943	11700	na	2
2011	na	69245	263323	166,000	na	na	19427	na

Sources:

Brazil, Ministry of Health, 2006;

AIDS data obtained from Brazil, Ministry of Health, National AIDS Program, 2011

Tuberculosis data (beginning 203): http://portal.saude.gov.br/portal/arquivos/pdf/casos_novos_tuberculose_1990_2011_16_02_2012_pub.pdf

Malaria data (beginning 2004): http://portal.saude.gov.br/portal/arquivos/pdf/tab_casos_confirmados_malaria_bra_gr_e_ufs_90a09.pdf

Dengue: http://portal.saude.gov.br/portal/arquivos/pdf/informe_dengue_2011_37_39.pdf

http://portal.saude.gov.br/portal/arquivos/pdf/boletim_semana_1_a_30_revisado_03_11.pdf

Years 2004-07: http://portal.saude.gov.br/portal/saude/deciframeoudevorote/files/ministerio/materiais/encontro_nacional/reuniaofaculdades.pdf

2011: http://crofsblogs.typepad.com/h5n1/2012/03/brazil-sees-66-drop-in-dengue-cases.html; http://en.mercopress.com/2012/04/25/rio-do-janeiro-admit-a-dengue-epidemic-over-50.000-cases-and-12-deaths

Hepatitis A/B (2004-2010): http://www.aids.gov.br/sites/default/files/anexos/publicacao/2011/50073/boletim_hepatites2011_pdf_64874.pdf

Meningitis, 2007: http://acritica.uol.com.br/noticias/Dados-Ministerio-Saude-Brasil-Amazonas_0_608939200.html

2009: http://portal.saude.gov.br/portal/arquivos/pdf/nt_meningite_brasil15_03.pdf

2011: http://portal.saude.gov.br/portal/saude/profissional/visualizar_texto.cfm?idtxt=37810

2009-2000: http://www.ripsa.org.br/fichasIDB/pdf/2010/FichaD1.16.pdf

Yellow Fever (2007-08): http://portal.saude.gov.br/portal/arquivos/pdf/boletim_svs_febre_amarela_040408.pdf

http://www.cve.saude.sp.gov.br/htm/zoo/FA_INFORME.htm

http://www.medicina.ufmg.br/rmmg/index.php/rmmg/article/viewFile/108/89

http://portal.saude.gov.br/portal/arquivos/pdf/boletim_febre_amarela_09_12_09.pdf

http://portal.saude.gov.br/portal/arquivos/pdf/tabela_1_fa_2010.pdf.

In response to international pressures and escalating TB cases throughout the 1990s, while the President and Ministry of Health (MOH) had already responded to TB by increasing their bureaucratic and policy commitment prior to the Global Fund’s emergence, the latter nevertheless helped to further deepen health governance at these multiple stakeholder levels while directly contributing to new community-based institutional innovations. Indeed, we found that the Global Fund helped strengthen national bureaucratic commitment to TB while incentivizing cooperation between previously uncooperative health agencies, such as the national AIDS and TB programmes, due mainly to the Global Fund’s grant requirement of addressing the TB-HIV/AIDS co-infection problem. At the same time, the Global Fund has helped to further increase inter-governmental cooperation between the National TB Programme (NTP) and sub-national municipal health agencies as well as between the NTP and non-governmental organizations (NGOs). Our findings also suggest that the Global Fund’s investments have not only helped deepen social mobilization, but it has also had a direct impact on fostering new institutional innovations at the community-level, thus further enhancing local participatory governance.

But as we discuss in the conclusion, it is important to note that the Global Fund has not had as positive of an impact in other nations. In contrast to Brazil, other countries simply do not have the same level of preexisting political and bureaucratic commitment, as well as the rich history of social mobilization in drawing attention to health issues and pressuring the government for policy reform. Consequently, despite the Global Fund’s presence, few nations have been able to see as positive of an impact on health governance.

## Materials and methods

In this study, we first conducted an extensive literature review discussing the impact that donor and global financial initiatives have on health governance. This was followed by empirical evidence from an in-depth case study of Brazil, which combined both qualitative and quantitative data from Global Fund grant disbursements to Brazil and MOH annual expenditures. We selected the case of Brazil for two key reasons: First, to provide a good example of how Global Fund investments can strengthen government and civil societal commitments and capacity to respond to relatively neglected diseases, such as TB, as well as increased coordination and accountability within and between government and civil society. Second, we selected the case of Brazil in order to provide greater knowledge, examples, and lessons about the complexities of health governance at multiple stakeholder levels.

Qualitatively, and as the Methodological Appendix explains, we conducted in-depth interviews with key informants from the Global Fund, national and sub-national health officials, medical doctors, academics, and activists. We conducted an iterative methodological approach [35]. Through this approach, researchers conduct the case study analysis during rather than after the collection of data [35]. By simultaneously and repeatedly consulting interview data with case study evidence from different phases of the analysis, we were able to develop and refine our initial research questions, identify causal patterns, devise concepts of health governance based on these patterns, and establish linkages between the data. This was done in order to corroborate our ideas and concepts while providing a more accurately defined case study. And in an effort to provide supportive evidence of our causal claims from multiple empirical sources, also known as the method of triangulation [36], which is useful when there is a limited amount of published findings, the interview data was supported with evidence from printed media, published articles, and reports. This study primarily took place from November 2009 to August 2010, with additional interviews undertaken in 2012.

## The impact of external investments on health governance

The benefits of integrating targeted health programmes into mainstream health system functions has been the subject of a longstanding debate in relation to the organization and financing of health systems focused on increasing access and improving health outcomes [37-39]. This debate has too often been fueled by polarized views of protagonists for and against the integration of targeted or vertical programs, arguing the relative merits of each approach [40]. This debate has been recently rekindled due to substantial increases in external investments for immunization programmes by GAVI, and AIDS, tuberculosis and malaria by the Global Fund. However, all too frequently the arguments for or against integration have not been underpinned by robust consistent evidence [17,41].

In the last decade, the major focus of G8 summits in Japan, Italy and Canada has been developing country approaches that foster both health systems strengthening and disease-specific targeted approaches. However, few studies have explored how and to what extent the new external financing and organisational arrangements provided by global financial initiatives, such as the Global Fund and GAVI, have influenced the governance of vertical health systems and horizontal programmes in countries receiving financing from these agencies [17].

This debate and lack of evidence highlights the importance of our research question: that is, how and to what extent does the Global Fund strengthen vertical and horizontal health governance approaches to disease? How do these external investments influence integration of vertical disease programmes, such as TB, into health systems and horizontal approaches, such as community participation through decentralisation processes, to addressing diseases? As the case of Brazil will illustrate, investments from the Global Fund can strengthen the integration of vertical programmes into health systems while strengthening community participation.

With regards to theory, we analyse the literature’s discussion of the impact that external investors, such as donors and global financial initiatives, have on health governance. While a number of recent studies have examined how political stewardship, accountability, inter-governmental accountability and cooperation, decentralisation, civic ownership, and participation affects the implementation of health policy [19,26,42,43], these studies limit their analysis to governance processes within governments, failing to address the impact that donors and global financial initiatives have on this process. With the exception of a recent study exploring how the Global Fund’s efforts to foster community ownership and participation have facilitated more inclusive policy development and implementation [5], little has been written on this process. Indeed, the few studies that examine donor and global financial initiatives' impact on health governance have focused on government and civil societal inclusion in the grant application process, either through the creation of formal decision-making mechanisms, such as the Global Fund’s Country Coordinating Mechanisms, or as explicit requirements for NGOs and other civic organisations seeking funding [44]; in essence, these are approaches which aim to empower civil society and build their trust and cooperation with the national government [4,45].

Other studies conducted by the World Bank and others exploring donor and global financial initiative impacts on health governance have emphasised their concerns with organisational and absorptive capacity to prudently manage external funds in a timely manner [2,7,10,12]. To strengthen local organisational and absorptive capacity, donors and global financial initiatives have simultaneously funded health systems and disease specific targeted programmes [21-24,46], as exemplified by the Global Fund, which by 2010 had approximately one-third of its approved investments in health systems strengthening, such as human resources (*e.g.*, employing doctors, nurses, healthcare workers and workforce training), health information systems, health financing, leadership and governance [11,33].

When analyzed from a political science perspective, however, these studies provide a limited view of global financial initiatives’ impact on health governance, as they do not address broader issues that are important for ensuring effective policy implementation, such as: (i) the impact global financial initiatives have on domestic government leadership and policy commitment – which is a key theme in the literature addressing the impact of donors on domestic AIDS politics and policy [14,47]; (ii) how global financial initiatives increase the interests of political and bureaucratic leaders in favour of addressing relatively neglected diseases, such as TB; (iii) how global financial initiatives’ support affects intra-bureaucratic relationships to promote multi-sectoral partnerships; and (iv) the extent to which these financial initiatives motivate national agencies to engage with state and municipal agencies as well as civil society when addressing health issues. Furthermore, while studies dealing with national-level processes have provided useful insight into the influence of widening stakeholder involvement on diversity and accountability in policy-making [4,45], they have not explored the way in which global financial initiatives have generated incentives to mobilise civil society, deepen their interaction with political and bureaucratic elite, and to promote greater transparency and accountability.

## Brazil

In contrast to the response to HIV/AIDS, when the Brazilian government created a national AIDS program and new prevention and treatment initiatives by the late-1980s, the government did not respond in a similar manner to TB. Brazil’s response to its TB epidemic was incongruous with the severity of the problem, which worsened as a result of increased urbanization, poverty, and HIV co-infection [48]. Throughout the 1980s and 1990s, moreover, the NTP did not have sufficient funding, both because TB was not perceived as an urgent public health threat and because of the economic recession, which impaired funding for most disease programmes [48,49]. The NTP, which was decentralised in the 1990s and consequently weakened [48,49], only began to receive political attention when new sources of funding became available.

Brazil first requested Global Fund financing in 2004 (Round 4 financing). Despite indicating a clear need for resources, Brazil’s request was initially declined by the Global Fund Technical Review Panel (TRP), mainly because of the Country Coordinating Mechanism’s (CCM) inadequate representation of civil society on the grant application [Global Fund official, personal communication, October 1 and 29, 2009; [48]. Nevertheless, in 2005 the request for Round 5 financing was recommended for approval by the TRP, as the revised CCM membership now included individuals affected by TB, other members from civil society as well as mechanisms ensuring the latter’s continued participation in policy-making [Global Fund official, personal communication, October 1 and 29, 2009; E. Santos-Filho, personal communication, June 30, 2006].

As Table 2 illustrates, US$23 million was approved by the Global Fund Board over five-years for the project "*Strengthening of the TB-DOTS Strategy in 10 Metropolitan Areas and in the City of Manaus in Brazil*," for two principal recipients (PRs): the Foundation for Scientific and Technological Development in Health (*Fundação para o Desenvolvimento Científico e Tecnológico em Saúde* - FIOTEC) and the Ataulpho de Paiva Foundation (*Fundação Ataulpho de Paiva -* FAP). The overall objective of the five-year grant was to expand DOTS coverage, increase social mobilization, information and awareness, reduce stigma, and to establish joint programs with the National AIDS Program [50]. By 2009, the PRs had received approximately $16 million of the approved funds and are scheduled to receive the remaining funds.

**Table 2:** Global Fund Grants

**TB – Round 5 (US$ millions)**			
Principle Recipients	Total Funding Requested	Approved Maximum	Total Funds Disbursed:
FAP/FIOTEC	$23, 021, 005.00	$23, 021, 005.00	$15,194,557.00
Total:	$23, 021, 005.00	$23, 021, 005.00	$15,194,557.00

Source: The Global Fund to Fight AIDS, TB, and Malaria, 2009.

Similar to what occurred with World Bank funding and HIV/AIDS policy in the early-1990s, the emergence of the Global Fund positively influenced and further accelerated the government’s preexisting commitment to responding to TB [48,49]. Prior to the Global Fund’s emergence in the 1990s, the rise of international criticisms and pressures for a stronger response to TB from international organizations, *e.g.*, the World Health Organization (WHO), and the media motivated the MOH to strengthen its bureaucratic and policy commitment to TB [48,51]. The MOH was essentially embarrassed with the government’s biased response to other communicable diseases, such as HIV/AIDS, and saw a renewed commitment to re-building the NTP as a way to rejuvenate the government’s reputation [48]. Moreover, by the early-1990s the number of TB cases had multiplied, thus making it impossible to ignore the situation. In response, in 1998 the MOH worked with the Congress to re-centralize the NTP’s policy responsibilities [48,51], such as monitoring TB cases, providing technical policy advice and guidelines, and funding medications [48,52,53]. The MOH also gradually increased its allocation of funding to the NTP to support these initiatives [48,52,53].

Nevertheless scholars soon began to identify the weaknesses of the NTP in effectively delivering prevention and treatment services [49,54]. Moreover, the NTP was not fully committed to working with local health departments and clinics [48]. Thus, while there was an increase in attention and resources for TB, national political and bureaucratic commitment to policy implementation was somewhat weak [48]. This was mainly due to the lack of sufficient funding, as well as motivation and accountability for the NTP’s actions.

The emergence of Global Fund support by 2006 nevertheless helped to strengthen the MOH’s pre-existing commitment to TB control [49]; D. Barreira, personal communication, October 20, 2009; F. Moherdaui, personal communication, June 16, 2006; V. Terto, personal communication, May 22, 2012; E. Santos-Filho, personal communication, May 23, 2012]. The need for additional financial resources as well as the government's newfound commitment to working with civil society as a condition for Global Fund support compelled the MOH to increase its commitment to the implementation of policies aimed at strengthening TB control [F. Moherdaui, personal communication, June 16, 2006; M. Dalcolmo, personal communication, October 20, 2006; B. Durovni, personal communication, July 7, 2006; V. Terto, personal communication, May 22, 2012; R. Burgos Filho, personal communication, May 23, 2012; Table 3. Additional funding from the Global Fund also provided the operational freedom and capacity for the NTP to appoint new staff (mainly from the national AIDS programme) while using Global Fund money to hire short-term consultants to work on the implementation of policies [C. Basilia, personal communication, November 15, 2009; M. Sanchez, personal communication, July 2, 2011; E. Santos-Filho, personal communication, May 23, 2012; R. Burgos Filho, personal communication, May 23, 2012]. Furthermore, in addition to further incentivizing MOH commitment to the NTP, Global Fund support motivated the President and the MOH to publicly discuss the NTP and its policies, at levels that were never before seen [48,55]; E. Santos-Filho, personal communication, June 30, 2006 and May 23, 2012; V. Terto, personal communication, May 22, 2012; R. Burgos Filho, personal communication, May 23, 2012].

**Table 3:** Brazil: available funding for the National TB Program

$US (millions)	2000	2001	2002	2003	2004	2005	2006	2007	2008	2009
	9.3	6.3	5.2	13.6	27.4	29.7	44.3	58.8	71.6	74

In Brazil, the Global Fund emerged at a time of bureaucratic discord within the MOH, with tensions between the AIDS and TB programmes dating back to the 1990s, due mainly to the biased support that the National AIDS Programme received [49]; A. Kritski, personal communication, July 20, 2006; M. Dalcolmo, personal communication, October 20, 2006; V. Terto, personal communication, May 22, 2012]. This conflict was sparked by the fact that the NTP had enjoyed strong financial support and visibility in the preceding decades [48]; M. Dalcolmo, personal communication, October 20, 2006; [49]. However, because part of the Global Fund’s grant money was to be used for addressing the rising TB-HIV/AIDS co-infection problem, TB and HIV/AIDS officials were motivated to strengthen their work together [Global Fund official, personal communication, October 1 and 29, 2009; D. Barreira, personal communication, October 20, 2009; Brazilian NTP official, personal communication, November 5, 2009; F. Moherdaui, personal communication, June 16, 2006; E. Santos-Filho, personal communication, May 23, 2012; R. Burgos Filho, personal communication, May 23, 2012]. Indeed, the Global Fund grant required that TB and AIDS officials develop new policy initiatives and awareness campaigns, such as conferences, in turn helping make the health problem more visible [ibid; [49]; G. Gerhardt, personal communication, July 19, 2006; F. Moherdaui, personal communication, June 16, 2006; V. Terto, personal communication, May 22, 2012; E. Santos-Filho, personal communication, May 23, 2012].

Coinciding with negotiations with the Global Fund to finalise contracts to receive funding, in 2005 the NTP created a special division, the *Coordenador Adjunto, Programa Nacional de Controle da Tuberculose* (Adjunct Coordinator of the National Tuberculosis Program), to strengthen its partnership with the National AIDS Programme [F. Moherdaui, personal communication, June 16, 2006; 50]. This was aided by the fact that the Adjunct Coordinator, Fabio Moherdaui, as well as the director of the NTP, Draurio Barreira, had previously worked in the National AIDS Programme and had extensive networks [V. Terto, personal communication, May 22, 2012; E. Santos-Filho, personal communication, May 23, 2012], experience, and a strong commitment to creating a collaborative partnership with the National AIDS Program [D. Barreira, personal communication, October 20, 2009; F. Moherdaui, personal communication, June 16, 2006; E. Santos-Filho, personal communication, May 23, 2012].

During this period, social mobilisation was also visibly profound and motivated by Global Fund financing for civil society. While previously no NGOs had been formally established to support TB control, in 2003 activists from the state of Rio formed the *Fórum Estadual das ONGs na luta contra a Tuberculose no Rio de Janeiro* (Forum of State NGOs against Tuberculosis in Rio de Janeiro); São Paulo followed suit with the creation of its own *Fórum* that same year, as well as the *Rede para o Controle Social da TB no Estado de São Paulo* (Tuberculosis Social Control Network of São Paulo) in 2005. These *Fórums* were comprised of community-based organizations, the church, businesses, sex worker organizations, feminist groups, as well as AIDS NGOs [48,56]. Activists and researchers nevertheless emphasize that the *Fórums* were not influential during this period in pressuring the NTP to strengthen its response to TB (C. Basilia, personal communication, October 17, 2006, and November 15, 2009; [48,56]. Indeed others have characterized the NTP’s relationship with the *Fórums* during this period as tenuous and superficial, a relationship that was created only to attract international financial support rather than to effectively include and increase the *Fórums’* policy ideas and influence within the NTP [56]. Nevertheless, the *Fórums'* size grew and were energized as the Global Fund-supported TB programmes rolled out – though it is important to note that the *Fórums* were never the principle recipients of Global Fund support [C. Basilia, personal communication, October 17, 2006, and November 15, 2009; [48,49]. The *Fórums* quickly began to mobilise in order to engage in the design and implementation of new policies [49]; C. Basilia, personal communication, November 15, 2009]. Because most of the policies that AIDS NGOs had pressured the government for, such as universal access to antiretroviral treatment (ARV), had been implemented by the late-1990s [32], there were fewer opportunities for AIDS NGOs to justify and receive funding to mobilize and work on these policy issues [57]. This prompted a number of relatively unemployed AIDS NGOs to join the *Fórums*[57,58], with the latter growing in size and in other cities [C. Basilia, personal communication, November 15, 2009; [48].

**Figure 2:**
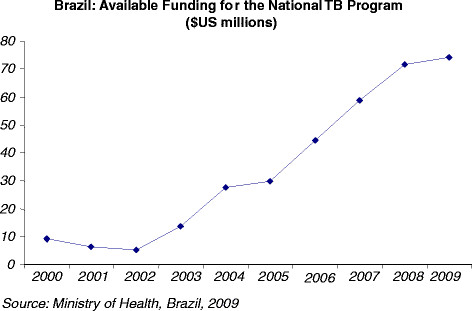
Brazil: National TB Program Funding available for activities with Civil Society.

In addition to forming a closer relationship with the NTP [M. Sanchez, personal communication, July 2, 2011; Figure 2; Table 4, the *Fórums* also drew greater attention to the HIV-TB co-infection problem, mainly through the media, workshops, and conferences [56]. *Fórums* have also worked closely with municipal and state health secretariats to organize national meetings to discuss policy issues, such as the *Encontro Comunitário das ONGs na Luta contra a Tuberculose no Estado do Rio de Janeiro* (Community Meeting of NGOs in the Fight Against Tuberculosis in the State of Rio de Janeiro).

**Table 4:** Brazil: National TB Program Funding available for activities with Civil Society

Reais $ (millions)	2007	2008	2009	2010
	536,492.00	726,718.00	1,501,819.00	1,162,000.00

Global Fund support has also helped strengthen inter-governmental cooperation between the NTP and sub-national governments – which is important, especially given the financial and administrative challenges associated with the devolution of health policy responsibilities through the *Sistema Único de Saúde* (SUS), the decentralised universal healthcare system [59]. Moreover, while the municipalities are responsible for collecting revenue and expenditures for healthcare [60], cooperation is important as the states and municipalities are reliant on the MOH for financial assistance through discretionary fiscal transfers [60,61], which are often conditional, based on the amount of responsibilities municipalities have agreed to take in providing healthcare services [62]. While inter-governmental cooperation did exist prior to the Global Fund’s establishment, beginning in 2002 to 2003 [E. Santos-Filho, personal communication, May 23, 2012], the NTP was not as committed to meeting with sub-national governments. Because of the Global Fund’s periodic assessment of CCM performance for grant renewal, the NTP had incentives to further increase its preexisting commitment to cooperating and meeting with state and municipal health departments [D. Barreira, personal communication, October 20, 2009; M. Sanchez, personal communication, July 2, 2011; E. Santos-Filho, personal communication, May 23, 2012]. The NTP has intensified its commitment to working closely with the states and municipalities in order to implement Global Fund financed projects [63]; D. Barreira, personal communication, October 20, 2009; M. Sanchez, personal communication, July 2, 2011; E. Santos-Filho, personal communication, May 23, 2012] as well as to provide adequate financial and technical assistance for FAP and FIOTEC’s work and DOTS implementation [64].

In addition, in order to ensure that principle recipients (PR) of grants have adequate support and work effectively, the NTP provides consistent technical support and site visits to discuss and evaluate policy implementation as well as needs [D. Barreira, personal communication, October 20, 2009; Brazilian NTP official, personal communication, November 5, 2009; M. Sanchez, personal communication, July 2, 2011; E. Santos-Filho, personal communication, May 23, 2012]. This technical support also helps to increase PR accountability to the NTP [M. Sanchez, personal communication, July 2, 2011]. The NTP also regularly interacts with municipal health secretaries in Rio and other cities to ensure that it has adequate technical support [ibid; V. Gallesi, personal communication, October 13, 2009; M. Sanchez, personal communication, July 2, 2011; E. Santos-Filho, personal communication, May 23, 2012]. Because the NTP is accountable, through the CCM, to the Global Fund for the FAP and FIOTEC's performance, the programme has further incentives to work closely with these institutions and the municipalities in order to ensure policy success [D. Barreira, personal communication, October 20, 2009; Brazilian NTP official, personal communication, November 5, 2009; V. Gallesi, personal communication, October 13, 2009; M. Sanchez, personal communication, July 2, 2011].

It is also important to note that the Global Fund has had a direct impact on health governance at the community-level, fostering a greater commitment to civic mobilization, empowerment, and accountability [V. Terto, personal communication, May 22, 2012; M. Sanchez, personal communication, July 2, 2011; E. Santos-Filho, personal communication, May 23, 2012]. In order to ensure that the PR's work effectively, in 2006 members of civil society in Rio and São Paulo, in conjunction with municipal health secretariats and the NTP, created *Comitês Metropolitanos* (Metropolitan Committees), which are independent entities, not financed by the Global Fund. Modeled after the 1988 constitutionally-mandated municipal health councils, these *Comitês* are public participatory institutions. AIDS NGOs, *Fórum* members, municipal health officials, people affected by TB, the church, and staff from the MOH’s Global Fund supported programme periodically meet to closely monitor the provision of Global Fund financing, review all aspects of Global Fund programmes, grant performance, impact, discussions for creating new funding proposals and potential PRs, as well as networking [63,65]; D. Barreira, personal communication, October 20, 2009; V. Gallesi, personal communication, October 13, 2009; L. Brilhante, personal communication, July 6, 2010; J. Matsudo, personal communication, August 17, 2010; A. Alegria de Almeida, personal communication, July 1, 2010; N. Faraone, personal communication, June 4, 2010; M. Sanchez, personal communication, July 2, 2011; E. Santos-Filho, personal communication, May 23, 2012].

Though initially created in Rio, *Comitês* have also emerged in eight other cities, namely Belo Horizonte, Fortaleza, Manaus, Porto Alegre, Recife, Salvador, São Luís, and São Paulo. Because of the high incidence of the TB-HIV co-infection, São Paulo has created two *Comitês*, which are well known for exhibiting a very strong commitment to civic participation [V. Gallesi, personal communication, October 13, 2009; N. Faraone, personal communication, June 4, 2010; A. Alegria de Almeida, personal communication, July 1, 2010].

These *Comitês* have provided even further opportunity for the NTP to work closely with civil society and local health departments [L. Brilhante, personal communication, July 6, 2010; J. Matsudo, personal communication, August 17, 2010; A. Alegria de Almeida, personal communication, July 1, 2010; N. Faraone, personal communication, June 4, 2010; V. Gallesi, personal communication, October 13, 2009; D. Barreira, personal communication, October 20, 2009; M. Sanchez, personal communication, July 2, 2011; E. Santos-Filho, personal communication, May 23, 2012]. The director of the NTP as well as MOH officials working on the Global Fund grant frequently attend *Comitês* meetings [ibid]; this demonstrates strong government support as well as oversight in the grant implementation process [66]; Brazilian NTP official, personal communication, November 5, 2009; D. Barreira, personal communication, October 20, 2009; V. Gallesi, personal communication, October 13, 2009; N. Faraone, personal communication, June 4, 2010; M. Sanchez, personal communication, July 2, 2011]. The *Comitês* also benefited from the direct support of the former Minister of Health, José Temporão, who attended meetings on a number of occasions [67].

The creation of municipal *Comitês* provides further evidence of the ongoing drive in Brazil to improve transparency and participatory governance for health. In essence, this community-level institution-building response is an extension of civil society's historic commitment to ensuring "social control" for health, the origins of which dates back to the 1960s with the *movimento sanitarista*[68]. This movement was committed to pressuring the government to provide universal healthcare as a constitutionally-guaranteed human right, socially controlled through the decentralization process [68].

**Figure 3:**
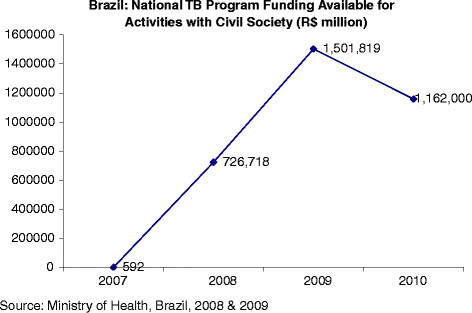
Brazil: Available Funding for the National TB Program.

Global Fund support has therefore had a positive impact on health governance in Brazil, engendering a stronger commitment to increasing engagement and participation at all levels of government and civil society [V. Terto, personal communication, May 22, 2012; E. Santos-Filho, personal communication, May 23, 2012]. Global Fund support has also provided new opportunities to deepen civic participation by directly involving TB victims and their supporters on the CCM. An analysis of recent budgetary allocations (Figures 1 and 3) suggests that civil society, including affected communities, has benefited from Global Fund support.

## Discussion

Our findings suggest that at the intra-governmental level, Global Fund financing has helped strengthen the increased engagement of the MOH in TB control, while fostering stronger cooperation between the national AIDS and TB programmes. At the inter-governmental level, Global Fund financing, its inclusiveness principle and accountability framework have also provided incentives for the NTP to strengthen its partnership with sub-national health departments and civil society. At the community-level, Global Fund support has also helped to deepen civic mobilization through the *Fórums*, expand civic participation in decision making, improve transparency and accountability, while fostering community-level institution-building with the creation of the *Comitês Metropolitanos*. These institutions have provided venues for strengthening collaboration between communities, the NTP, and local health officials.

However, it is important to note that the Global Fund was limited in its direct contribution to the creation of multiple levels of health governance in Brazil. While the Global Fund did directly contribute to the creation of community-based accountability institutions (*i.e.*, the *Comitês Metropolitanos*), it did not instigate national government interest and commitment to responding to TB. Rather, Global Fund financing complemented and further strengthened pre-existing efforts to address TB at the national government level (E. Santos-Filho, personal communication, May 23, 2012).

Furthermore, it is important to note that it was not the simultaneous combination of Global Fund money *and* civil societal pressures that gave the MOH the confidence and incentives needed to strengthen its response to TB. While Global Fund financial support was necessary, civil society’s pressures for policy reform through the *Fórums* were present prior to the Global Fund’s emergence; however, these civic pressures did not have any influence on strengthening the MOH’s commitment to policy reform [17,48,56]. The influence of the *Fórums* and civil society on strengthening the MOH’s commitment to policy reform only seemed to emerge after the Global Fund mandated civil society’s inclusion onto the CCMs and PRs. Therefore it seems that Global Fund support and its grant conditionalities, not civil societal pressures, were the most important factors leading to a further increase in national government responses to TB.

Several key lessons emerge from analyzing the Global Fund’s impact on health governance in Brazil. First, while empirical research has explored health governance and policy implementation in developing countries [26,36,42,65,69], as well as its impact on stake-holder inclusion, transparency, and financial management [4,7,10,12,45], few have examined the impact that global financial initiatives have on multiple levels of health governance. The case of Brazil shows that a more nuanced analysis of health governance at multiple levels provides a more robust demonstration of the extent to which global financial initiatives further compel national politicians, bureaucrats, and civil society to respond to relatively neglected diseases, while building new institutions and accountability mechanisms at the community level.

Second, this case study suggests that Global Fund support can foster participatory approaches to addressing relatively neglected diseases, such as TB, at levels that are similar to country responses to HIV/AIDS [3,69].

Third, the availability of new funding also increased the attention and support of previously marginalized national TB officials, motivating them to work in a more collaborative manner with national AIDS officials and civil society in response to TB. Thus by funding the NTP to address the TB/HIV co-infection problem, the Global Fund has also helped catalyse and strengthen inter-agency cooperation for a more aggressive response to TB.

Fourth, an interesting new finding relates to institution-building at the community-level. Earlier studies on health governance have explored the construction of effective *national-level* institutions, such as national AIDS programmes and commissions [14,70]. In Brazil, financing from the Global Fund amidst the backdrop of pre-existing political and constitutional commitments to social control over health policy can motivate local communities to work with local governments in creating new community-based institutions, such as the *Comitês Metropolitanos*. These community-based institutions are created with the expressed intent of deepening civic engagement, accountability, and social control. Whereas national AIDS commissions have served this purpose at the national-level, *Comitês* have shifted governance and engagement to the community-level. While Brazil may be unusual in this regard, given its rich history of social control for health, future work will need to explore the extent to which communities in other nations are building new institutions to ensure the effective use of international funding.

Given the financial crisis faced by the Global Fund and its policy direction to withdraw from middle-income countries, the durability of the Global Fund’s positive effects in Brazil are in question. Furthermore, after the Global Fund denied Round 9 funding to the NTP in November 2009 [71], on the grounds that the government had sufficient revenue to finance its TB programmes, there have been concerns that the NTP and civil society will remain committed to working together in implementing policies once Global Fund financing ceases. However, so far it seems that sustaining this commitment and partnership between the NTP and civil society will occur, and for two reasons: first, the government and civil society’s preexisting commitment to responding to TB, and second, the Global Fund’s impact in further strengthening MOH commitments to policy implementation, as well as creating new venues for civil society to further strengthen its mobilization and monitoring capabilities, such as the *Comitês Metropolitanos*, which continue to thrive [E. Santos-Filho, personal communication, May 23, 2012]. Notwithstanding the inability to obtain further Global Fund support, recent interviews with activists and government officials suggests that the government and civil society is still equally if not more committed to working together in order to curtail the spread of TB [E. Santos-Filho, personal communication, May 23, 2012; V. Terto, personal communication, May 22, 2012; R. Burgos Filho, personal communication, May 23, 2012; M. Sanchez, personal communication, July 2, 2011; D. Barreira, personal communication, June 10, 2011; P. Werlang, personal communication, August 1, 2011].

Nevertheless, it is important to note that the Global Fund's impact in other nations has not been as positive. In China, for instance, research finds that the provision of Global Fund grants for AIDS prevention and treatment in 2004 did not lead to inter-bureaucratic coordination, increased coordination between the MOH and the states, as well as the creation of new community-based institutions [72]. While the Global Fund's presence did increase civil societal participation on CCMs, many NGOs were controlled by the MOH, while selection processes for CCM participation were politically manipulated until very recently [73]. In contrast to Brazil, the absence of a rich history of social movements and participation in health policy-making and political accountability for policy implementation complicated the Global Fund's ability to deepen health governance in China.

Other countries have experienced similar challenges [74]. In the Ukraine, despite being a PR for Global Fund assistance, the MOH did not have the political commitment needed to manage the grant money effectively [74]. Consequently, when the Global Fund recommended that the MOH no longer be a PR, MOH officials objected and negatively reacted by withdrawing their support for the Global Fund’s work with AIDS NGOs [74]. This revealed the Ukrainian MOH’s self interest and unwillingness to reform its managerial funding procedures in order to be re-considered as a PR, while lacking the commitment needed to ensure that AIDS NGOs’ needs were met. Similarly, in Myanmar, during the mid-2000s the MOH was not committed to helping AIDS and TB NGOs work with Global Fund staff, such as denying Global Fund staff entry visas to visit programme sites [74]. This revealed the government’s lack of commitment in facilitating the PR recipients’ work, which in turn suggested the MOH’s apathy in ensuring civil societal needs.

Finally, in other countries, such as Cambodia and the Cameroon, NGOs have not had the organizational, technical, financial managerial, and communication skills needed to work effectively with health officials and to hold them accountable [38]. Moreover, NGOs on CCMs in these nations have little experience in mobilizing and organizing committees outside of the CCM in order to hold it and the Global Fund accountable [38]. In this context, and in sharp contrast to Brazil, the Global Fund’s influence in deepening health governance and accountability has been rather limited.

Because of these challenging environments, others have viewed the Global Fund’s investments as causing more harm than good. In addition to providing new opportunities for government corruption as a result of CCM mandates, as seen in China, others have been critical of the consequences associated with the Global Fund’s decision to suddenly discontinue grants, often due to allegations of corruption and grant mismanagement in recipient nations [75], as well as the Global Fund’s lack of transparency in decision-making [74,75]. In some cases the permanent or temporary cancellation of grants has delayed the work of NGOs working on prevention and treatment programmes [75]. In Indonesia, for example, several healthcare workers resigned from positions with PRs because of the non-payment of salaries and financial uncertainty [75]. Most of these criticisms of the Global Fund can also be attributed to the limitations in governments’ commitment to closely monitoring PRs and CCMs, government commitment to transparency, eradicating corruption, and ensuring that PRs and CCMs can work freely with Global Fund staff.

What this therefore suggests is that the generalizability of our findings in Brazil may be limited to the case of Brazil; this gives the impression, moreover, that Brazil is *sui generis* in terms of having the propitious, preexisting political commitment and history of effective social health movement mobilization and accountability [73]. These preexisting conditions have facilitated the Global Fund’s positive affects at multiple levels of health governance. Future research will need to examine if other countries have similar preexisting conditions and if not, what else the Global Fund can do to help strengthen this process.

We acknowledge that there are some limitations to our study. First, there are no earlier studies on the subject, thus scant empirical evidence to draw on. Our study addresses this problem by conducting extensive interviews and triangulating our interview data with other types of empirical evidence, while engaging in an iterative methodological process. Second, the argument could be made that electoral incentives as well as the historic legacy of progressive government commitment to combating epidemics and social control would have eventually led to an aggressive response to TB. While we did not address these issues, to our knowledge, no national- or community-level politician has ever campaigned on the TB issue, while though present, a very limited national and community-based response occurred prior to the Global Fund's emergence [48,56]. Third, the influence of other lenders, donors, and global financial initiatives needs to be taken into account when reaching conclusions. For example, while the World Bank has been helpful in stimulating national- and community-based responses to AIDS [8,32], while the USAID and Management Sciences for Health (MSH) has provided funding for TB prior to the Global Fund in order to improve the provision of drug treatment through DOTS [48], neither was specifically focused on strengthening health governance at multiple stakeholder levels. To our knowledge, aside from the Global Fund, no other donor or global financial initiative has focused on this issue.

## Conclusion

This study has emphasized the potentially positive affects that global financial initiatives have on strengthening multiple levels of health governance. Considering the world-renowned success of the Brazilian government’s response to AIDS and its progressive commitment to universal healthcare, it is interesting that Brazil approached the Global Fund for help in its response to TB. Doing so nevertheless revealed that in the absence of external financial support and accountability to the Global Fund, own its own the MOH may not have responded as aggressively to TB, nor would civil society have had a reason to become more innovative in creating new accountability institutions at the community level.

While we have built on the work of recent scholars emphasizing the need to expand our concepts and analysis of health governance to the governmental and community-level [25,76], our work highlights the importance of examining how global financial initiatives can further strengthen this process. Indeed, while the MOH’s commitments to responding to TB was present prior to the Global Fund, and while social movements, such as the *Fórums*, had emerged by 2003, we found that the Global Fund’s investments in Brazil helped to further strengthen health governance within government and civil society. More specifically, the provision of additional funding, when combined with the Global Fund’s mandate that the MOH address the TB-HIV/AIDS co-infection problem and work closely with civil society, deepened health governance at the intra- and inter-governmental level, while leading to new community-based institutional innovations.

Findings from Brazil further suggest that the Global Fund’s success in strengthening health governance at multiple levels is ultimately determined by antecedent historical political and social conditions. Global Fund staff and other international health financiers need to better understand the political and social context of the nations that they are working in, rather than mechanically focusing on the management of grant finances. Context matters: that is, undertaking contextual and health system analysis is critical to ensuring desired health policy outcomes at a time of increasingly scarce international resources for health.

## Appendix

Methodological appendix - interviews

The individuals interviewed for this study were chosen from officials working in the Global Fund to Fight AIDS, Tuberculosis, and Malaria, the Brazilian National TB Programme (NTP), municipal tuberculosis officials, members of civil society participating in the *Fórums, Comitês Metropolitanos*, as well as individuals not working in the TB sector, such as AIDS NGO leaders, activists, national AIDS officials, and academics. Extending our interviews to the non-TB sector provided a balanced, unbiased view of the issues that we were interested in. Those Global Fund individuals chosen to be interviewed were selected based on their direct participation in Global Fund support to Brazil and policy formulation at the Global Fund. Those senior officials interviewed at the Global Fund asked to remain anonymous. Two interviews were conducted in October 1, 2009, and October 29, 2009. In Brazil, national and municipal tuberculosis program officials were selected based on their leadership in implementing TB policy, working with the Global Fund (mainly national officials) as well as the PRs (Principal Recipients) and *Comitês* implementing Global Fund policy. In addition, members of the *Comitês* in several cities were interviewed because of their experience working with the NTP and municipal health officials. Finally, those individuals not working in the TB sector were chosen for their knowledge of the TB sector, the NTP’s interaction with the Global Fund, and personal impressions of the Global Fund’s impact on health governance in Brazil. None of those interviewed for this study were chosen for their a-prior policy views and beliefs, thus allowing us to avoid the usage of biased interview data.With regards to NTP officials, the following individuals were interviewed: the director of the NTP, Draurio Barreira, October 20, 2009 and June 10, 2011 (30 minute phone interview); Patricia Werlang, NTP official, August 1, 2011 (email survey); a senior official within the NTP working on the Global Fund grant, November 5, 2009 (30 minute phone interview); and Fabio Moherdaui, NTP, June 16, 2006. With regards to municipal health officials, Vera Gallesi, coordinator of the São Paulo state TB program, October 13, 2009 (by phone); Margaret Dalcolmo, Reference Center for Tuberculosis, Rio de Janeiro, October 20, 2006; Germano Gerhardt, President, *Fundação Ataulpho de Paiva*, Rio de Janeiro, July 19, 2006; Bettina Dorovni, director of the division of AIDS, TB, and Colera, municipal department of health, Rio de Janeiro, July 7, 2006 (by phone).Members of civil society and activists included: Afranio Kritski, *Universidade Federal do Rio de Janeiro*, July 20, 2006; Carlos Basilia, director, *Fórum Estadual das ONGs na Luta contra a Tuberculose no Rio de Janeiro*, November 15, 2009 and October 17, 2006 (phone interview); Ezio T. Santos-Filho, Vice President, *PellaVida* NGO, Rio de Janeiro, June 30, 2006; members of the Global Fund *Comitês Metropolitanos* included: Nadja Faraone, São Paulo, June 4, 2010 (by phone); Joyce Matsudo, Manaus, Amazonia, August 17, 2010 (email survey); Laíze Brilhante, Recífe, July 6, 2010 (email survey); Ana Cristina Alegria de Almeida, Costa da Mata Atlantica, São Paulo, July 1, 2010 (email survey).Those non-TB individuals that were interviewed included AIDS NGO leaders, activists, national AIDS officials and university professors: Veriano Terto, Executive Director of *ABIA*, Rio de Janeiro, May 22, 2012 (30 minute phone interview); Ezio T. Santos-Filho, former Vice President of *PellaVida* AIDS NGO, Rio de Janeiro, currently PhD Candidate, Medical School, Federal University of Rio de Janeiro, May 23, 2012 (30 minute phone interview); Ruy Borgos Filho, Director, National AIDS Program, Brasilia, May 23, 2012 (30 minute phone interview); and Mauro Sanchez, Adjunct Assistant Professor, *University of Brasilia*, Brasilia, July 2, 2011 (30 minute phone interview).

## Competing interests

The authors have no competing interests to declare.

## Authors' contributions

EG and RA are the primary authors of this study, having devised the theoretical and empirical analysis. All authors read and approved the final manuscript

## Ackowledgements

We wish to acknoweldge KH for his research assistance. No funding was obtained for this study.
